# Participation in organised sports does not slow declines in physical activity during adolescence

**DOI:** 10.1186/1479-5868-6-22

**Published:** 2009-03-31

**Authors:** Mathieu Bélanger, Katherine Gray-Donald, Jennifer O'Loughlin, Gilles Paradis, Jennifer Hutcheon, Katerina Maximova, James Hanley

**Affiliations:** 1 Centre de formation médicale du Nouveau-Brunswick, Université de Moncton and Université de Sherbrooke, Moncton, Canada; 2 Beauséjour Research Centre, Regional Health Authority A, Moncton, Canada; 3 Department of Epidemiology, Biostatistics and Occupational Health, McGill University, Montreal, Canada; 4 School of Dietetics and Human Nutrition, McGill University, Montreal, Canada; 5 Department of Social and Preventive Medicine, Université de Montréal, Montreal, Canada; 6 Centre de recherchedu Centre Hospitalier de l'Université de Montréal, Montreal, Canada; 7 Institut national de santé publique du Québec, Montreal, Canada; 8 Research Institute of the McGill University Health Centre, Montreal, Canada

## Abstract

**Background:**

Among youth, participation in extracurricular physical activities at school and organised physical activities in the community is associated with higher physical activity levels. The objective was to determine if participation in organised physical activities during early adolescence protects against declines in physical activity levels during adolescence.

**Methods:**

Every 3 months for 5 years, students initially in grade 7 (aged 12–13 years) completed a 7-day physical activity recall and provided data on the number and type of (extracurricular) physical activities organised at school and in the community in which they took part. To study rates of decline in physical activity, only adolescents who reported an average of ≥5 moderate-vigorous physical activity sessions per week in grade 7 (n = 1028) were retained for analyses. They were categorised as to whether or not they were involved in organised physical activities in grade 7. We used generalized estimating equation Poisson regression to compare the rate of decline in number of moderate-vigorous physical activity sessions per week during adolescence between initially physically active students who participated in organised physical activity in grade 7 and those who did not.

**Results:**

In grade 7, about 87% of physically active adolescents reported taking part in at least one organised physical activity. Compared to active adolescents not involved in organised physical activities, baseline involvement in physical activity was 42% (95% CI 26–59%) higher among those involved in organised physical activity (mean number of moderate-vigorous physical activity sessions per week = 14.6 ± 13.1 vs 10.4 ± 9.0). Physical activity declined by 8% per year in both groups. Results were similar in analyses that examined the effect of school or community-based physical activities separately.

**Conclusion:**

Although participation in organised physical activities during early adolescence is associated with more physical activity throughout secondary school, participation in such activities does not protect against declines in physical activity over time.

## Introduction

Despite emerging evidence that physical activity improves longevity and quality of life,[1,2] the majority of people in industrialized countries are physically inactive [3,4]. Among Canadian adolescents, less than one in ten are sufficiently physically active to meet current recommendations on physical activity[5] and most have declining levels of physical activity during adolescence [6]. Given that physical inactivity in youth is one of the best predictors of a sedentary lifestyle in adulthood,[7] there is a need to identify activities that will help adolescents maintain healthy activity levels.

Extracurricular physical activities at school and organised physical activities in the community provide important opportunities for adolescents to be physically active[8,9] and contribute to helping young people attain recommended physical activity levels [10,11]. However, it is unclear if participation in organised physical activities in early adolescence protects against declining physical activity levels later during adolescence. We hypothesised that because of factors such as the structure and social support that are associated with organised physical activities, these activities could contribute to maintaining healthy physical activity levels among their participants. Previous studies suggested that adolescents prefer structured physical activities [12,13] and that social support for physical activity is associated with better sustainability of healthy activity levels [14,15]. The objective of this analysis was to determine if participation in organised physical activities at school and in the community during early adolescence influences physical activity participation during secondary school.

## Methods

### Study population

Data were obtained from the Nicotine Dependence in Teens study, a prospective study of 1293 students initially 12–13 years old. Participants were recruited from grade 7 classes in a convenience sample of 10 Montreal-area secondary schools. Schools were selected to include a mix of urban, suburban, and rural schools, as well French and English language schools located in high, moderate, and low socioeconomic neighbourhoods. Thirteen schools were initially invited to participate; and while all schools agreed, two were excluded because of a low return rate of consent forms and one because school administrators could not guarantee cooperation over the entire study. Over half (55.4%) of eligible students entered the study; the relatively low response was related to the need for blood sampling for genetic analysis and to a province-wide labour dispute that resulted in some teachers refusing to collect consent forms. Subjects and a parent or guardian provided assent and signed informed consent, respectively. Data collection began in the 1999–2000 school year and was repeated every 3 months for 5 years, for a total of 20 survey cycles (the first survey cycle was administered between October and January). Because of very little potential for decline in physical activity participation, and given that the aim of this analysis was to compare rates of decline between groups of initially physical active adolescents, participants who were physically inactive in the first year of study were excluded from the analyses (see data analysis section for definition of physically active and inactive). Participants completed questionnaires at school, in the language of instruction of the school. The study received approval from the McGill University Institutional Review Board.

### Study variables

The outcome studied was the number of sessions of moderate or vigorous physical activity per week. This was obtained from a seven-day physical activity recall similar to those used in other large-scale studies [16,17]. The question was worded: "Think about the physical activities that you did last week from Monday to Sunday outside your regular school gym class. For each activity that you did for 5 minutes or more at one time, mark an "X" to show the day(s) on which you did that activity...," followed by a list of 29 activities, including 23 that were of moderate or vigorous intensity (i.e., activities with an estimated energy cost over 4.8 metabolic equivalent values[18]). Respondents checked which activities they had engaged in on each day of the previous week. The 3-day test-retest reliability of the original instrument was *r *= *0.74*[19] and our version of the physical activity recall showed evidence of convergent validity with energy intake [20].

Participation in organised extracurricular physical activities at school was measured by: "Since September of this school year, did you belong to any of the following intramural or extramural school sports teams (teams that were not part of your regular gym class)...?" This was followed by a list of 13 sports teams. Participants were categorized as "no" organised school physical activities if they did not check any of the 13 school teams during the first year of study (i.e., in the four survey cycles completed in grade 7). They were categorized as "yes" if they indicated that they had participated on any of the 13 school teams in at least one of the grade 7 four survey cycles.

Similarly, participation in extracurricular physical activities in the community was measured by: "Now think about sports teams and lessons outside of school. In the past 3 months, did you belong to a...? (followed by a list of 12 possible sports teams or instructor led physical activities)". Participants were categorized as "no" organised community physical activities if they did not check any of the 12 community physical activities when in grade 7. They were categorized as "yes" if they had participated in any of the 12 community physical activities.

The two categorisation schemes described above were combined to categorize participants as "no" participation in any organised physical activities or as "yes" if they had participated in at least one of the two forms of organised physical activities in grade 7.

Data on covariates including age and sex, were drawn from the baseline questionnaire. Because climate variations influence physical activity through the year [21], season (categorized into fall (September to November), winter (December to February), and spring (March to June)), was also included as a covariate. No data were available for the summer months since data collection occurred only during the 10-month school year.

### Data analyses

As indicated earlier, only participants who were categorised as physically active during the first year of study were retained for these analyses. Participants were categorised as active if they reported an average of 5 or more moderate or vigorous physical activity sessions per week in grade 7. Analyses conducted with other cut-points (3, 4, 6, 7) to categorise participants as physically active or not had the same results.

We used Poisson regression analyses to assess if there were differences in the rate of physical activity decline from grade 7 to grade 11 between adolescents who participated in organised physical activities in grade 7 and those who did not. Three models were developed which investigated the effect of participation in organised physical activities at school, in the community, and in "any" organised physical activities separately. Analyses were conducted within the generalized estimating equations framework to account for non-independence of repeated observations in individuals. Clustering at the school level was taken into account by using an indicator variable for each school. We tested interaction terms between time and the dummy variables indicating participation in organised physical activity (at school, in the community, or both) in grade 7. Because estimated effects were similar for boys and girls in sex-specific analyses, we present results for the overall sample with sex as a covariate. Also, analyses including all participants (physically inactive and active), where we controlled for baseline physical activity level (covariate for baseline activity level and an interaction term between baseline level and time) yielded similar findings as the models presented herein. Analyses in which grade 8 (instead of grade 7) involvement in extracurricular activities was used to categorize participants also produced similar results. Finally, the conclusions derived from the models presented herein are the same as the ones associated with sensitivity analyses in which the exposure of interest (participation in organised physical activities) was expressed as continuous variables (the most parsimonious models were retained in the interests of simplicity of interpretation). We used the SAS statistical package version 9.1 GENMOD procedure with Poisson distribution for modeling purposes and used a two-sided Cochran-Armitage test for trend to assess the trend in the prevalence of participation in organised activities over five years (SAS Institute Inc, Cary, NC).

## Results

Grade 7 data on physical activity were available for 1276 participants (17 joined the cohort after grade 7 and were not retained for this analysis). These participants took part in a median (interquartile range) of 18 (11 to 19) of 20 survey cycles. Approximately 94% of eligible participants completed questionnaires at each follow up. Reasons for non-participation in a survey cycle included: moved to a non-participating school (71%); dropped out of the study (17%); absent on the day of data collection (11%); and other (1%). There were no meaningful or statistically significant differences in sex or baseline physical activity level between participants involved in the study for 1–4 years, and those who participated for the full five years. The mean number of moderate or vigorous physical activity sessions at baseline was 14 (median 10). About 20% (n = 248) of participants were categorised as "physically inactive" in the first year of study and were not retained for the following analyses. Of participants retained, 53% and 82% reported participation in physical activities organised at school and in the community in grade 7, respectively (Table 1).

**Table 1:** Descriptive characteristics of the study population (n = 1028)

Involvement in organised physical activity in grade 7, n (%)	
Organised extracurricular physical activity at school	
No	482 (46.9)
Yes	546 (53.1)
Organised physical activity in community	
No	181 (17.6)
Yes	847 (82.4)
Any organised physical activity	
No	132 (12.8)
Yes	896 (87.2)
No. physical activity sessions/week in year 1, mean ± SD	
Organised extracurricular physical activity at school	
No	15.7 ± 10.4
Yes	17.9 ± 12.5
Organised extracurricular physical activity in community	
No	11.8 ± 6.6
Yes	17.7 ± 12.1
Any organised extracurricular physical activity	
No	12.5 ± 6.9
Yes	17.5 ± 12.0
Age at baseline, *mean *± *sd *(years)	12.8 ± 0.6
Sex (n, % female)	531 (51.7)

Among participants involved in organised physical activities at school, 58%, 24%, 9%, and 9% reported being members of one, two, three, or four or more school teams in grade 7, respectively. With regards to participants in community physical activities, 36%, 31%, 14%, 11%, and 8% reported involvement in one, two, three, four, and five or more of such activities, respectively. The most frequently reported activities were soccer, basketball, and volleyball for school activities and soccer, swimming, and hockey for community activities. The correlation between the number of moderate or vigorous physical activity sessions per week and the number of organised physical activities involved in at school and in the community in grade 7 was weak (Spearman *r *= 0.1 and 0.2, respectively).

In univariate analyses, the number of physical activity sessions engaged in per week was, on average, 39% (95% confidence interval (CI): 24 to 57%) higher among participants in organised physical activities (mean ± standard deviation = 14.6 ± 13.1) than among physically active adolescents who were not involved in organised physical activity when in grade 7 (10.4 ± 9.0). Participation in extracurricular physical activity organised at school in grade 7 (14.9 ± 13.4 vs 13.1 ± 11.7) was associated with 14% (CI: 4 to 23%) higher levels of physical activity across secondary school, whereas participation in physical activity organised in the community in grade 7 (14.8 ± 13.1 vs 9.5 ± 8.7) was associated with reporting 52% (CI: 37 to 70%) more physical activity sessions per week. The decrease in the number of moderate or vigorous physical activity sessions per week averaged 3% (CI: -2 to -3%) per survey cycle and resulted in an overall 40% decrease in number of moderate or vigorous physical activity sessions per week by the end of the 5 years of follow up. Every year, study participants engaged in an average of 1.4 fewer physical activity sessions per week.

The estimated effect of organised physical activity participation remained the same after adjustments for age, sex, season, and school (Table 2). The rate of decline in number of moderate or vigorous physical activity sessions engaged in per week was equivalent in the two groups of adolescents categorised as active in grade 7 (involved in organised physical activities vs not). The models that examined the effect of school and community-based physical activities separately presented similar results. Those who participated in organised physical activities in grade 7 maintained an average of four more physical activity sessions per week than active adolescents not involved in such activities during early adolescence. Only less than 2% of the variance in frequency of participation in moderate or vigorous physical activity sessions was explained by the variable representing participation in organised physical activities. Although girls' initial level of physical activity was lower than that of boys, the differences in rate of physical activity decline between those involved in organised physical activities and active individuals not involved in such activities were similar in both sexes (data not shown). Further analyses indicated that the proportion of participants reporting involvement in any organised physical activities decreased from 87% to 79%, 72%, 70%, and 59% for each of grades 7 to 11, respectively (*p *< 0.0001).

**Table 2:** Adjusted percent difference in number of moderate or vigorous physical activity sessions per week according to participation in organised physical activities (n = 1028)

	**Model for organised physical activity at school**		**Model for organised physical activity in the community**		**Model for any organised physical activity**	
	
**Variable**	**Difference**^a, b^	**95% CI**	**Difference**^**a, b**^	**95% CI**	**Difference**^**a, b**^	**95% CI**
Organised physical activity						
No	Reference		Reference		Reference	
Yes	↑ 16%	6, 26%	↑ 46%	31, 63%	↑ 42%	27, 59%
Time (survey cycle)^c^	↓ 3%	-3, -2%	↓ 3%	-4, -2%	↓ 2%	-3, -1%
Interaction terms						
Time × No	Reference		Reference		Reference	
Time × Yes	↔	-1, 1%	↔	-1, 1%	↓ 1%	-2, 0%
Age at baseline (years)	↓ 1%	-8, 7%	↔	-7, 8%	↔	-7, 7%
Sex (female)	↓ 25%	-30, -19%	↓ 24%	-29, -18%	↓ 24%	-30, -19%
Season						
Fall	Reference		Reference		Reference	
Winter	↓ 13%	-15, -11%	↓ 13%	-15, -11%	↓ 13%	-15, -11%
Spring	↑ 10%	7, 12%	↑ 10%	7, 12%	↑ 10%	7, 12%
Intercept (95% CI)	25.8 (9.6, 69.4)		17.1 (6.3, 46.3)		18.3 (6.7, 49.6)	

CI, confidence interval; ^a ^Also adjusted for school;^b ^Percent difference in number of moderate or vigorous physical activity sessions per week derived from Poisson regression rate ratio. Alternatively, the percent difference for participation in organised physical activity at school (category "Yes" in model for school physical activity) can be interpreted as a rate ratio of 1.16 with 95% CI = 1.06 to 1.26. ^c ^Time unit is the 3-month interval between survey cycles; ↑, increase; ↓, decrease; ↔, no effect.

## Discussion

Similar to other studies, [22-24] the average frequency of physical activity participation among adolescents in this study declined with age. Involvement in organised physical activity during early adolescence, whether at school or in the community, did not influence the rate of decline in physical activity. However, participation in organised physical activity, particularly community activities, was associated with more frequent participation in moderate or vigorous physical activity throughout adolescence.

Although it was recently reported that organised physical activities are more popular among adolescents than unorganised activities,[12,13] organisation around physical activity does not seem to prevent a decline in participation. Factors that are commonly reported to explain discontinuation of physical activity in adolescents, such as lack of time, employment, changing priorities or interests, and "doing like their friends", [25-27] may be relevant to both organised and unorganised physical activities. Similarly, organised and unorganised physical activities may be equally related to maintenance or improvement in fitness, the most commonly reported benefits of physical activity among adolescents [26]. Adolescents may therefore maintain or discontinue participation in physical activities that are organised and others that are not for similar reasons.

In this study, we restricted our analyses to adolescents who were physically active in the first year of study. The observed difference in frequency of physical activity participation between adolescents involved in organised physical activity during early adolescence and other initially active adolescents not involved in such activities was therefore not confounded by the low likelihood that initially inactive adolescents be involved in organised physical activities. Consequently, our results support previous studies which did not control for initial physical activity level but reported that participation in organised sports during early adolescence is associated with more physical activity later on [28,29]. This suggests that the promotion of organised physical activities in early adolescence could help youth maintain healthier activity levels throughout secondary school, even though it will not prevent declines.

To interpret our results appropriately, some methodological limitations should be considered. First, participants in this study were not randomly selected. However, participants were similar to a representative sample of Canadian youth with regards to the prevalence of participation in organised physical activities [30]. Second we did not have access to an objective measure of physical activity. Because each activity session of five minutes or more were counted, it is possible that activity levels were overestimated. In other cases, it is possible that activity levels were underestimated if the checklist did not capture certain types of activities. However, the repeated use of a physical activity checklist, as in this study, generally provides a valid estimation of the level of involvement in moderate or vigorous physical activity [31].

## Conclusion

In summary, this study indicates that participation in organised physical activity at school or in the community during early adolescence helps students maintain higher levels of physical activity participation during the secondary-school years. However, it does not appear to protect against declining physical activity levels during adolescence. Adolescents should nevertheless continue to be encouraged to enrol in organised physical activities since it contributes to higher levels of physical activity.

## Competing interests

The authors declare that they have no competing interests.

## Authors' contributions

MB conceived the objectives of the analysis, designed and carried out statistical analyses, interpreted the data, and wrote the manuscript. KGD, JOL, GP, JH, KM, and JH contributed to data interpretation and reviewed the manuscript critically for important intellectual content and writing. JOL designed the original study, obtained the funding, and supervised the data collection.
